# Improved subseasonal prediction of South Asian monsoon rainfall using data-driven forecasts of oscillatory modes

**DOI:** 10.1073/pnas.2312573121

**Published:** 2024-04-01

**Authors:** Eviatar Bach, V. Krishnamurthy, Safa Mote, Jagadish Shukla, A. Surjalal Sharma, Eugenia Kalnay, Michael Ghil

**Affiliations:** ^a^Department of Environmental Science and Engineering, California Institute of Technology, Pasadena, CA 91125; ^b^Department of Computing and Mathematical Sciences, California Institute of Technology, Pasadena, CA 91125; ^c^Center for Ocean-Land-Atmosphere Studies, George Mason University, Fairfax, VA 22030; ^d^Fariborz Maseeh Department of Mathematics and Statistics, Portland State University, Portland, OR 97201; ^e^Department of Atmospheric and Oceanic Sciences and Institute for Physical Science and Technology, University of Maryland, College Park, MD 20742; ^f^Department of Atmospheric, Oceanic and Earth Sciences, George Mason University, Fairfax, VA 22030; ^g^Department of Astronomy, University of Maryland, College Park, MD 20742; ^h^Geosciences Department and Laboratoire de Météorologie Dynamique (CNRS and Institut Pierre-Simon Laplace), École Normale Supérieure and Paris Sciences et Lettres University, Paris, France 75005; ^i^Department of Atmospheric and Oceanic Sciences, University of California, Los Angeles, CA 90095; ^j^Department of Mathematics, Imperial College London, London SW7 2BX, United Kingdom

**Keywords:** South Asian monsoon, ensemble forecasting, data-driven forecasting, subseasonal-to-seasonal prediction

## Abstract

Predicting the temporal and spatial patterns of South Asian monsoon rainfall within a season is of critical importance due to its impact on agriculture, water availability, and flooding. The monsoon intraseasonal oscillation (MISO) is a robust northward-propagating mode that determines the active and break phases of the monsoon and much of the regional distribution of rainfall. However, dynamical atmospheric forecast models predict this mode poorly. Data-driven methods for MISO prediction have shown more skill, but only predict the portion of the rainfall corresponding to MISO rather than the full rainfall signal. Here, we combine state-of-the-art ensemble precipitation forecasts from a high-resolution atmospheric model with data-driven forecasts of MISO. The ensemble members of the detailed atmospheric model are projected onto a lower-dimensional subspace corresponding to the MISO dynamics and are then weighted according to their distance from the data-driven MISO forecast in this subspace. We thereby achieve improvements in rainfall forecasts over India, as well as the broader monsoon region, at 10- to 30-d lead times, an interval that is generally considered to be a predictability gap. The temporal correlation of rainfall forecasts is improved by up to 0.28 in this time range. Our results demonstrate the potential of leveraging the predictability of intraseasonal oscillations to improve extended-range forecasts; more generally, they point toward a future of combining dynamical and data-driven forecasts for Earth system prediction.

## Significance

The South Asian monsoon affects more than a billion people in the Indian subcontinent. The monsoon intraseasonal oscillation (MISO) determines the spatial structure of the monsoon rainfall on subseasonal timescales, and its accurate prediction is therefore key for agricultural and hydrological planning. Here, we combine data-driven forecasts of MISO with an ensemble of dynamical forecasts of the full system, leveraging the predictability of MISO to improve monsoon forecasts. Our results show significant improvement compared to state-of-the-art dynamical model forecasts, demonstrating the potential of data-driven forecasts to improve subseasonal monsoon prediction.

The South Asian monsoon is one of the most important seasonal features of the global climate system. Despite modest improvements in recent years, the forecast skill of monsoon prediction has lagged behind improvements in numerical weather prediction (1–3). Moreover, accurate forecasts of regional rainfall on intraseasonal timescales, rather than just the seasonal mean rainfall, are crucial for the agricultural and hydrological sectors (4).

## Monsoon Intraseasonal Variability

It has long been known that the monsoon possesses intraseasonal variability in the form of active and break phases within the summer monsoon season (5–7). The active phase is associated with high rainfall over central India, while the break phase is associated with low rainfall over central India, but high rainfall over northern and southeastern India (6).

These intraseasonal variations are dominant at two spectral peaks, roughly at 45 d and 20 d (8). They consist of northward-propagating rainfall anomalies, referred to as monsoon intraseasonal oscillations (MISOs), closely tied to the boreal summer intraseasonal oscillation (BSISO) (9). MISOs characterize the active and break phases of the monsoon and much of the regional rainfall patterns (8).

Current state-of-the-art dynamical models poorly predict MISOs (2, 10, 11). Improving MISO prediction is recognized as a crucial part of improving monsoon forecasts on intraseasonal timescales (1).

## Data-Driven Forecasts

Oscillations in the climate system, due to their near-regularity and low frequency compared to synoptic-scale weather variability, are recognized as an important source of predictability beyond the weather timescale (12). This recognition has led to work in predicting these oscillatory modes using data-driven methods.^*^

There is an extensive literature on data-driven forecasting of climate oscillations (13), and several works have developed data-driven (10, 14, 15) or low-order model (9, 16) forecasts for MISO. Some of these methods demonstrate skill in MISO prediction up to 50 d, while dynamical models only demonstrate skill for up to 30 d, and often less (17–19). The superiority of data-driven forecasts may be due to model error in the dynamical forecasts, but can also be associated with initial condition error (20). This gap demonstrates the potential for improved intraseasonal prediction of monsoon rainfall.

Since the intraseasonal oscillations only comprise a fraction of the total variance of the field of interest, their prediction is not directly useful (21–24). For instance, MISOs comprise about 14% of the variance in daily rainfall anomalies over India (8), or about 23% of the variance of 15-d rainfall over the monsoon region (25). Moreover, there is no way to faithfully infer the state of the full field from a forecast of an oscillatory mode (13). This suggests, therefore, the need for a method that combines the information from a full dynamical model with that of the data-driven forecasts.

## Prediction Using Dynamical and Data-Driven Forecasts

The idea of combining full-field dynamical forecasts with data-driven forecasts of intraseasonal oscillations was previously suggested by various studies (15, 21, 22, 26, 27). While there have been a number of works that correct dynamical forecasts of an oscillatory mode using either data-driven forecasts (28–30) or post-processing (31–33), these did not attempt to correct the full-field forecasts. In this work, we correct a full-field dynamical forecast using data-driven forecasts of specific modes.

Strong (21) was perhaps the first to propose a method for correcting the physical model forecasts, by adding a term nudging the dynamical equation to the statistical forecast, or by using a Kalman filter.^†^ However, to our knowledge, this was never implemented. Recent work (34–36) suggested a multi-model data assimilation–based approach for similar problems.

**Fig. 1.**
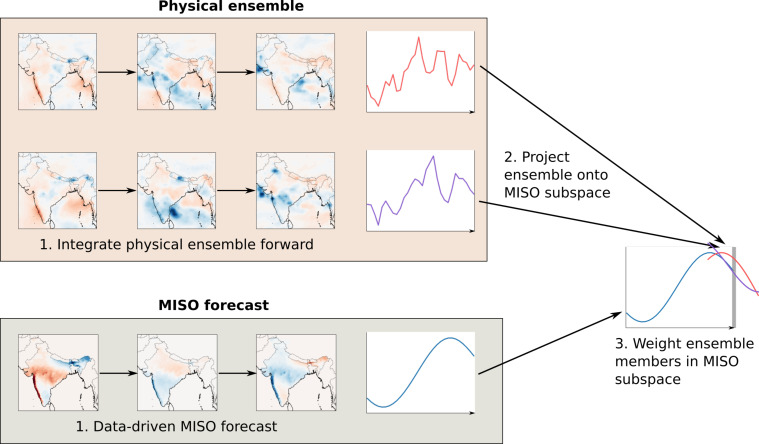
A simplified diagram of the EnOC algorithm, with two dynamical ensemble members for simplicity. Here, the second (purple) ensemble member will receive a higher weight, since it is closer to the MISO forecast in the subspace. Note that in the real implementation, we reduce the dynamics in the MISO subspace to the first two principal components of the MISO mode. See *SI Appendix*, section 1A for more details.

In Bach et al. (13) we introduced Ensemble Oscillation Correction (EnOC), to beneficially combine data-driven forecasts of oscillations with an ensemble of dynamical forecasts of the full system. EnOC works by projecting a dynamical ensemble into a subspace corresponding to the mode of interest, and weighting the ensemble members by their distance from a data-driven oscillation forecast in that subspace. See Fig. 1 for a basic schematic, ref. 13, and *SI Appendix*, section 1A for more details.

Here, we apply EnOC to the South Asian monsoon by leveraging the predictability of MISO. Our approach possesses two crucial features of machine learning applied to weather and climate problems (37, 38): 1) It is interpretable, in that the forecast improvements can be attributed to a specific physical mode; and 2) it does not introduce spurious unphysical features, since the forecast is always the mean of a subset of the dynamical ensemble. An additional advantage of EnOC is that in the current implementation, it is carried out offline, meaning that it is applied as a post-processing step to model outputs, avoiding the need for access to the computational and data resources of operational forecasting centers.

## Results

### Data-Driven MISO Forecast Skill.

**Fig. 2.**
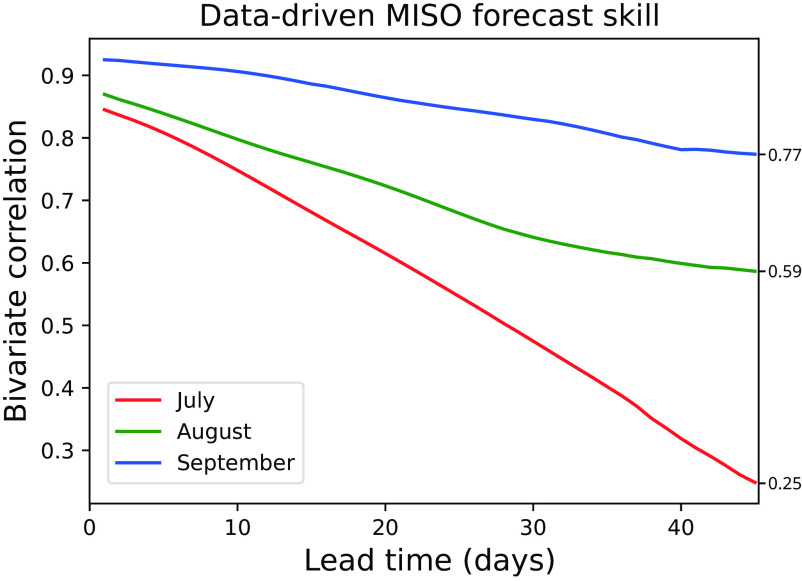
Correlation coefficient between predicted and observed MISO mode as a function of lead time, for forecasts starting on the 1st of July, August, and September, from 2008 to 2016.

First, we quantify the prediction skill of the data-driven MISO forecasts by comparing these to the MISO mode extracted from observations. We use the bivariate correlation coefficient, defined in *SI Appendix*, section 5A. As per Fig. 2, these forecasts maintain a bivariate correlation above 0.5 for over 46 d when initialized in August and September, and for about 28 d when initialized in July. The higher forecast skill in the later months is consistent with (9, 39). The reason for this phenomenon should be investigated in future research.

Some of the previous works on MISO prediction (10, 15) did not test the real-time forecasting context, in which one has to deal with filtering end effects. This filtering is the reason why the bivariate correlation coefficient in Fig. 2 does not begin at 1 at a lead time of 0. These results can be compared to ref. 9 and achieve similar skill.

### Improvements in Precipitation Forecasts.

We now focus on the improvements in the full-field precipitation forecasts made through application of EnOC. In the following results, we use the 15-d mean rainfall for the skill calculations: That is, at lead time i, we compare the mean rainfall forecasted over leads i−7,i−6,…,i+7 to the observations averaged over the same interval. Due to the highly intermittent nature of rainfall, such smoothing is often used in the literature (40, 41). We also include the results with 7-d averaging, which are qualitatively similar, in *SI Appendix*, section 6E.

We show results both over India and the wider monsoon region, which we define as 6–39°N and 66–100°E. The latter includes India, Bangladesh, Bhutan, Kyrgyzstan, Myanmar, Nepal, Sri Lanka, Tajikistan, most of Pakistan, southwestern China, and part of the northern Indian Ocean. This domain is close to the extended Indian monsoon rainfall region (42) widely used in studies on the South Asian monsoon.

We use the temporal correlation to quantify the skill of rainfall forecasts. For a given initialization time and lead time, we compute the correlation of the predicted to observed total rainfall over a given spatial domain, over the verification period of 2008 to 2016.

#### All-India and Monsoon Region.

**Fig. 3.**
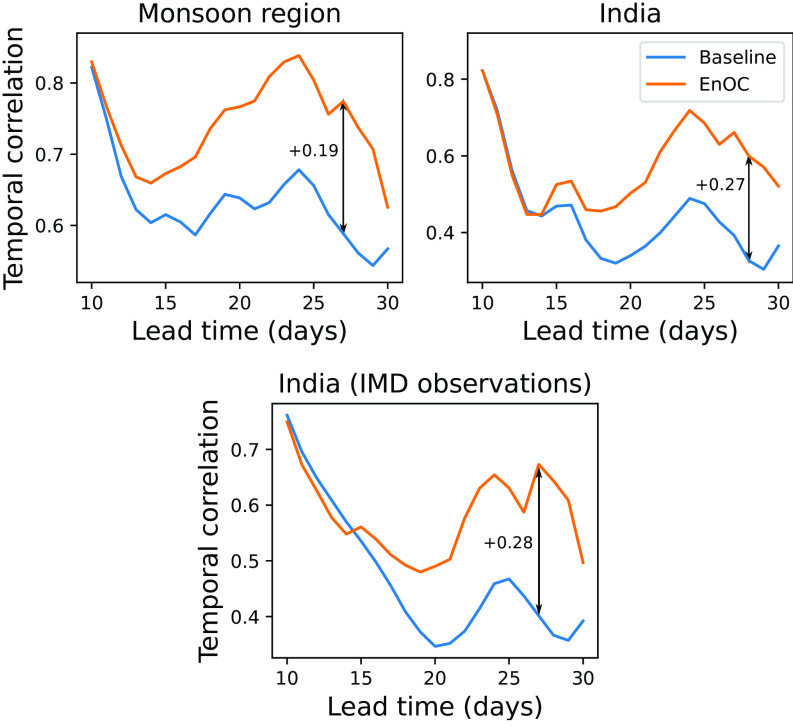
*Top*: Temporal correlation of monsoon region and India rainfall averages over forecasts initialized in July and August, verified against ERA5 reanalysis. *Bottom*: Same as *Top Right*, but verified against India Meteorological Department (IMD) observations.

We consider the temporal correlation of rainfall averaged over India and the wider monsoon region. We achieve a remarkable improvement in this correlation using the data-driven MISO forecast for July and August, as shown in Fig. 3. In July, the improvement in the temporal correlation reaches over 0.3 in the 23 to 28 d range over the monsoon region. Over India, the improvement reaches over 0.15 in the 22 to 30 d range. In August, the improvement over the monsoon region reaches 0.09, and over India reaches over 0.1.

Although there is no apparent improvement in the temporal correlation for September, there is improvement in other skill metrics discussed below. The average improvement for July, August, and September forecasts over the monsoon region is statistically significant at the 95% confidence level for the total 10 to 30 d rainfall, using the bootstrap method discussed in *SI Appendix*, section 7.

#### Regional results.

**Fig. 4.**
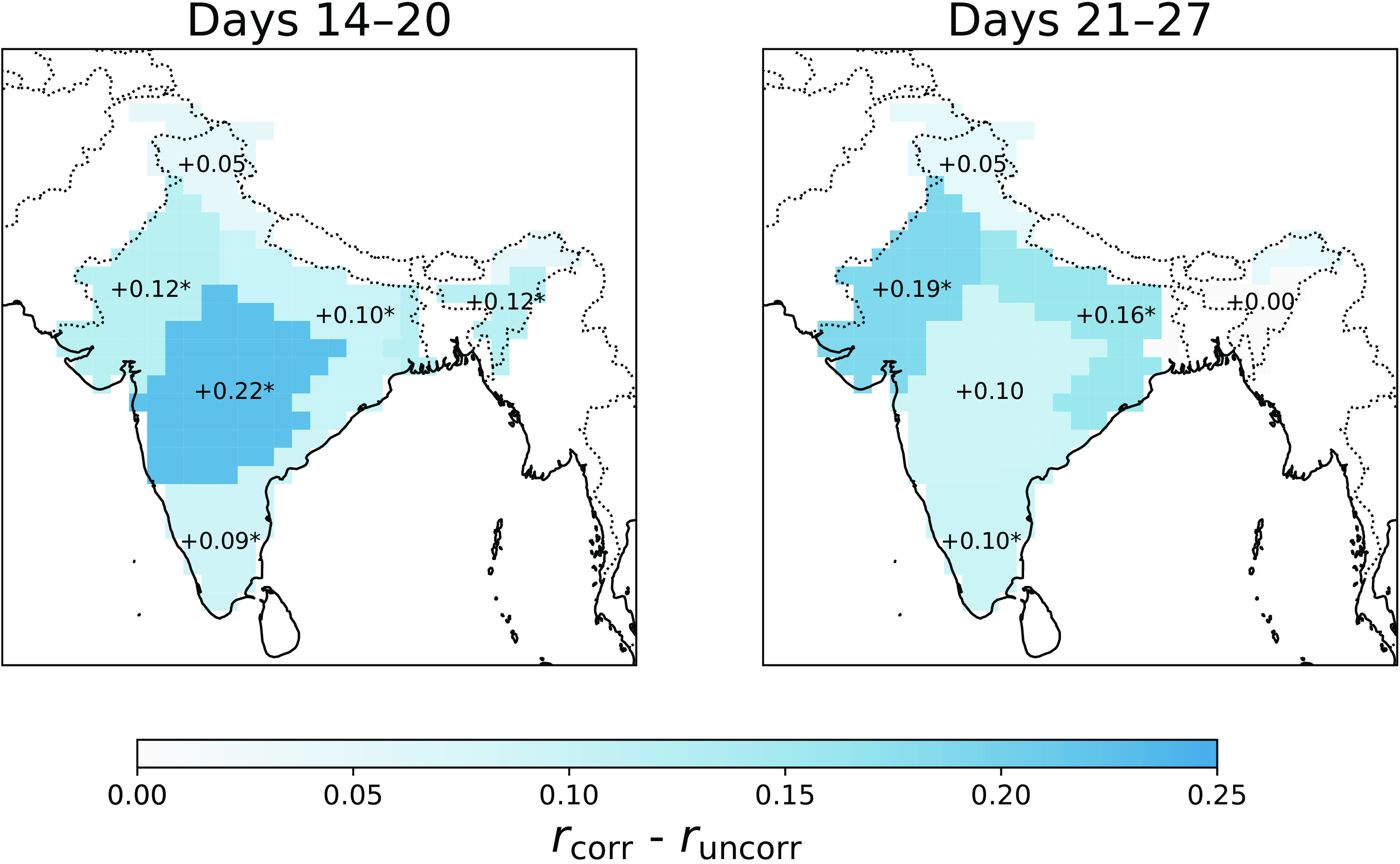
The difference between the corrected and uncorrected temporal correlation of forecasted rainfall by region. The asterisks indicate statistical significance at the 85% confidence level using a bootstrap methodology described in *SI Appendix*, section 7.

We now look at the temporal correlation of rainfall in the homogeneous rainfall regions of India; see Fig. 4. *SI Appendix*, section 3 defines the regions.

For 14- to 20-d lead time forecasts, there are statistically significant skill improvements (at the 85% confidence level) in all regions except for Hilly Regions, with the largest improvements in the West Central (+0.22), Northwest (+0.12), and Northeast (+0.12) regions. For 21- to 27-d lead time forecasts, the skill improvements are statistically significant in the Northwest (+0.19), Central Northeast (+0.16), and South Peninsular (+0.10) regions.

#### Other skill metrics.

In *SI Appendix*, section 6, we evaluate the forecasts with respect to three other skill metrics: the anomaly correlation, RMSE skill score, and the skill in predicting the MISO index defined in ref. 25. The EnOC forecasts are also improved with respect to these skill metrics.

#### Consistency of improvements in precipitation forecast skill.

To verify that the improvements in precipitation prediction are indeed due to improved prediction of MISO, we compute the correlation between 1) the difference between the errors in the dynamical and data-driven predictions of the MISO mode and 2) the difference in the RMSE of uncorrected and corrected precipitation forecasts. If the improvements in the EnOC-corrected forecasts of precipitation were due to the skill of the data-driven MISO forecast, we would expect a positive correlation. Indeed, over forecasts initialized in July, August, and September, and lead times from 8 to 39 d, the correlation is 0.37. For the individual months, we have July (0.29), August (0.52), and September (0.45). For all these correlations, the two-sided P-value is <0.001, with a null hypothesis of no correlation.

As another verification of the consistency of the forecast skill improvement, we examine the forecasts of low-level (850 hPa) relative vorticity. MISO modulates relative vorticity over the monsoon region: in active phases, the vorticity is increased, which in turn increases the genesis of strong low-pressure systems and precipitation (43–45). Due to this physical link, one would expect the EnOC-corrected ensemble to also have better prediction of relative vorticity, despite the fact that the data-driven forecast was based only on precipitation. *SI Appendix*, Section 6F shows that this is indeed the case: the EnOC ensembles have improved temporal correlation over India as well as improved anomaly correlation over the monsoon region.

## Conclusion

The MISO is predictable beyond the predictability limit of chaotic weather variability, due to its regularity and low frequency (10). Harnessing this predictability is an important part of improving monsoon prediction on intraseasonal timescales. We show that data-driven MISO forecasts can significantly improve state-of-the-art dynamical precipitation forecasts in the South Asian monsoon region. The improvements in the temporal correlation reach 0.15 to over 0.25 on 10- to 30-d lead times.

This methodology could also be applied to other intraseasonal oscillations, such as the Madden–Julian oscillation, for which data-driven forecasts are quite skillful (46–48). More generally, this work demonstrates the power of combining dynamical and data-driven models for Earth system prediction. Other recent work on such hybrid forecasting has also been promising (49, 50), as has the growing field of purely data-driven full-field forecasts on subseasonal-to-seasonal timescales (50–52). As machine learning forecasts of weather and climate continue to improve, we envision the integration of dynamical and data-driven forecasts for both real-time prediction and data assimilation. Recent work on multi-model ensemble Kalman filters (35) provides a versatile method for this application.

## Materials and Methods

### Data.

We use the IMD gridded rainfall dataset (53) for extraction and prediction of MISO. It is based on interpolation of rain gauge data and is provided at 0.25°×0.25° resolution with daily coverage from 1901 to 2016.

Unless otherwise stated, we use the ERA5 reanalysis as verification for forecasts. ERA5, and its predecessor ERA-Interim, is generally the most accurate in precipitation among reanalyses produced by major operational centers and is accurate in the South Asian monsoon region in particular (54–56). We also verify against the IMD rainfall observations in Fig. 3.

### Extraction of MISO.

We extract MISO from the IMD rainfall data using multi-channel singular spectrum analysis (M-SSA: 57, 58). M-SSA applies principal component analysis to multivariate delay-embedded time-series data, in order to identify and extract spatiotemporal modes. More information is provided in *SI Appendix*, section 1B.

M-SSA has previously been used to extract MISOs in a number of studies, from precipitation, outgoing longwave radiation, and wind fields (8, 10, 44, 59–63). Krishnamurthy and Shukla (8) demonstrated the existence of two MISO modes, with periods of about 45 d and 20 d, extracted from precipitation data. Here, we focus on the 45-d northeastward-propagating mode, which is most prominent, and shown to be statistically distinguishable from red noise in ref. 10. We closely follow refs. 8 and 10 in the extraction of the mode, and *SI Appendix*, section 4 provides some of its characteristics.

Following ref. 10, we furthermore take the two leading spatial empirical orthogonal functions (EOFs) of the MISO reconstructed component, and henceforth consider only the dynamics of the corresponding two-dimensional principal component (PC) time-series.

### Projection onto the MISO subspace and data-driven forecasts.

The process of reconstructing a mode using M-SSA involves forward and reverse filtering in time, posing a problem for real-time forecasting of the mode (9, 13, 64). In this real-time forecasting context, we must approximate the projection onto the MISO subspace, necessarily incurring an error in the initial conditions used for the data-driven forecast.

Here, we use a simple neural network architecture (described in *SI Appendix*, section 1C) in order to project from the full phase space onto the two-dimensional MISO space. To obtain the initial condition for the data-driven MISO forecasts (step 1 in Fig. 1), we use the current day and past 28 d of IMD rainfall data as inputs to the network. To project the dynamical ensemble members onto the MISO space (step 3), we use the current day, past 14 d, and 14 future days of forecasts for each ensemble member. For lead times less than 14 d, we use the current day and 28 future days.

We follow refs. 10 and 65 in the data-driven prediction of the MISO mode. We look for the closest historical analogs in the two-dimensional MISO space and average over their trajectories at a given lead time. See *SI Appendix*, section 1D for more details.

Note that EnOC does not depend on a specific data-driven forecasting method. Future work could use other methods for approximating the projection (13, 64), or use a method designed for real-time forecasting, where an approximate projection does not need to be used.

### Dynamical Forecast System.

We apply EnOC to hindcasts from SEAS5, the European Centre for Medium-Range Weather Forecasts (ECMWF) seasonal forecasting system (66). The atmospheric component is the Integrated Forecasting System (IFS) model, with a configuration similar to ECMWF’s operational medium- and extended-range forecasts, and NEMO is used for the ocean. We use hindcasts from 1993 to 2016, initialized on the first of July, August, and September, which are provided with 25 ensemble members. Note that the first 2 wk of June are an interval of rapid change during the monsoon onset, which provides a plausible explanation for the fact that data-driven forecasts do not exhibit satisfactory skill when initialized on June 1st. We have not included them, therefore, in the forecasts reported herein. The ECMWF forecasts have been shown to outperform those of other operational centers in predicting Asian monsoon intraseasonal variability (17, 18, 67), making them a state-of-the-art baseline.

We perform a simple bias correction of SEAS5 by subtracting its own climatology dependent on lead and initialization time from 1993 to 2007. This practice is common in subseasonal-to-seasonal forecasting (68). The years for bias correction did not overlap with the verification period of 2008 to 2016, avoiding the problem of artificial skill (69). The years 1993 to 2007 are also used for tuning the $m^{\prime }$ parameter of EnOC for each initialization time and lead; see *SI Appendix*, section 1A.

## Supplementary Material

Appendix 01 (PDF)

## Data Availability

The raw and processed forecasts, as well as the code for the method have been deposited in Zenodo (70).

## Footnotes

^*^Here, we use “data-driven” to mean that the forecasts are accomplished entirely by learning from past data. Data-driven forecasts are also referred to in the literature as statistical, empirical, or machine learning forecasts.

^†^Ideas attributed to Eugenia Kalnay and Robert Vautard, respectively.
