# Supplementary material for: Improved subseasonal prediction of South Asian monsoon rainfall using data-driven forecasts of oscillatory modes
Source: Proc Natl Acad Sci U S A. 2024 Apr 1;121(15):e2312573121. doi: 10.1073/pnas.2312573121 (PMC11009656; doi:10.1073/pnas.2312573121)
Supplement: Supplementary file 1 — Appendix 01 (PDF) [file pnas.2312573121.sapp.pdf]

Supporting Information for

Improved subseasonal prediction of South Asian monsoon rainfall using data-driven forecasts of oscillatory modes

Eviatar Bach, V. Krishnamurthy, Safa Mote, Jagadish Shukla, A. Surjalal Sharma, Eugenia Kalnay, and Michael Ghil

Eviatar Bach.
E-mail: eviatarbach@protonmail.com

This PDF file includes:

Supporting text
Figs. S1 to S13
SI References

Supporting Information Text

1. Methods

A. Ensemble Oscillation Correction (EnOC). We implement Ensemble Oscillation Correction as described below, based on (1). The projection and data-driven oscillation forecast methods differ from those used in (1), and are described in sections C and D below.

To apply EnOC to a dynamical ensemble forecast starting at time t_i for a lead time ℓ with ensemble size m, we:

1. Input the IMD observations at times $[t_i - 28, \ldots, t_i]$ into the neural network described in section C, obtaining an approximate point $\mathbf{r}_{t_i}$ in the MISO subspace.

2. Using $\mathbf{r}_{t_i}$ as the initial condition, forecast to time $t_i + \ell$ as described in section D, obtaining $\tilde{\mathbf{r}}(t_i + \ell)$ as the data-driven MISO forecast.

3. For each ensemble member of the dynamical forecast, input the forecasts at lead times $[t_i + \ell - 14, \ldots, t_i + \ell + 14]$ into the neural network described in section C, obtaining m points $\{\mathbf{r}^1, \ldots, \mathbf{r}^m\}$ in the MISO subspace.*

4. Compute the Euclidean distances $d_j = \|\tilde{\mathbf{r}}(t_i + \ell) - \mathbf{r}^j\|$ for $j = 1, \ldots, m$.

5. For the EnOC forecast, keep the dynamical ensemble members corresponding to the m' smallest distances d_j.

m' is optimized over the training period for each initialization day (July 1st, August 1st, and September 1st) and each lead time to minimize the RMSE against ERA5. When the time-averaged results are shown, we take the intersection of the sets of ensemble members picked at the leads $i - 7, i - 6, \ldots, i + 7$. This ensures that averaging is done over a continuous time-series, and is found to improve the results by exploiting the autocorrelation in the precipitation.

B. Multi-channel singular spectrum analysis (M-SSA). We briefly introduce M-SSA here; for more details, see (2). M-SSA applies principal component analysis (PCA) to portions of a multivariate time series obtained via sliding windows, identifying the spatiotemporal modes that capture the most variance in the time series.

Suppose we have a time series in D variables of length N, $\mathbf{x} = \{x_d(n) \,|\, d = 1, \ldots, D; \, n = 1, \ldots, N\}$. We must choose an embedding length M based on the time scales of the modes we are interested in capturing (3). We then create the time-lag embedded time series by forming $\mathbf{x}_d(n) = [x_d(n), \cdots, x_d(n + M - 1)]$ for each d, with $n = 1, \ldots, N - M + 1$. Then, we form the trajectory matrix by concatenating the $\mathbf{x}_d(n)$, $\mathbf{X} = (\mathbf{x}_1, \cdots, \mathbf{x}_D)$, and create the covariance matrix $\mathbf{C} = \mathbf{X}^T\mathbf{X}/(N - M + 1)$.

The covariance $\mathbf{C}$ has eigenvectors $\{\mathbf{e}_k\}$ with corresponding eigenvalues $\{\lambda_k\}$. The eigenvectors are called space-time EOFs (ST-EOFs) (2). The eigenvalues provide information about the variance captured by their ST-EOF: the ratio $\lambda_k / \sum_j \lambda_j$ is the fraction of the total variance captured by mode k. As in Fourier analysis, oscillatory modes appear as pairs of eigenvectors with eigenvalues that are nearly the same (2).

To extract the portion of the time series corresponding to mode k, we carry out the reconstruction procedure as follows. First, the trajectory matrix is projected onto the eigenvector $\mathbf{e}_k$:

$$\mathbf{a}_k = \mathbf{X}\mathbf{e}_k. \tag{1}$$

The reconstructed component (RC) at time n, for mode k, and dimension d, can be written as

$$r_{dk}(n) = \frac{1}{M_n} \sum_{m=L_n}^{U_n} a_k(n - m + 1) e_{dk}(m), \tag{2}$$

where $L_n = 1$, $U_n = M$, and $M_n = M$, except near the start and end of the time series, for which the appropriate values are given in (3). Summing over all the RCs recovers the original time-series, while summing over a subset yields a partial reconstruction (2). In the case of an oscillation, we will present the sum of the RCs corresponding to the eigenvalue pair.

Here, we apply M-SSA to the IMD rainfall dataset with $M = 61$. We only use March to November in M-SSA. This means that the M-SSA is performed on non-contiguous "multi-trial" time-series data, which we stack together.

In order to more efficiently compute the eigendecomposition of $\mathbf{C}$ for this high-dimensional system, we use the "transpose trick" often used in the PCA literature. This is advantageous when $DM \gg N - M + 1$. That is, we use the fact that if $\mathbf{v}$ is an eigenvector of a matrix $\mathbf{A}^T\mathbf{A}$, then $\mathbf{A}\mathbf{v}$ an eigenvector of $\mathbf{A}\mathbf{A}^T$. This means that the $(N - M + 1) \times (DM)$ trajectory matrix $\mathbf{X}$ can be replaced by $\mathbf{X}^T$ in the computation of the covariance matrix $\mathbf{C}$, resulting in an $(N - M + 1) \times (N - M + 1)$ matrix instead of $(DM) \times (DM)$. Then, the eigenvectors of the original matrix can be recovered by multiplying them by $\mathbf{X}$. See also section A2 of (2).

*If $\ell < 14$, we take $[t_i, \ldots, t_i + 28]$ instead. See section C.

C. Neural network for projecting from the full phase space onto the oscillation subspace. Here, we use neural networks to project from the full phase space to the oscillation subspace, both to obtain the initial condition for a MISO forecast as well as to obtain the state in the MISO subspace corresponding to a forecast. Due to model error, we train separate networks for these tasks. We use a 4-layer neural network, implemented in TensorFlow (4); Fig. S1 shows the architecture. The network uses ReLU activation functions and is trained using the Adam optimizer.

The neural network for projecting the initial conditions at time t onto the MISO subspace is trained on windows of $[t-28,\ldots,t]$ of IMD observations as input and the MISO PCs at time t as output. For projecting the dynamical forecasts initialized at time t and with lead time ℓ onto the MISO subspace, we train on ERA5 reanalysis as input and the MISO PCs as output. We train two separate networks: one for leads $\ell \geq 14$, for which we use the window $[t+\ell-14,\ldots,t+\ell+14]$ as input and the PCs at time $t+\ell$ as output, and one for leads $\ell < 14$ for which we use the window $[t+\ell,\ldots,t+\ell+28]$ as input and the PCs at time $t+\ell$ as output.

Instead of neural networks, other regression approaches can be used as well. For example, we found that ℓ_1-regularized linear regression, the lasso, works almost as well.

D. Data-driven MISO forecasts. We follow a similar procedure to (1, 5) in implementing the MISO forecasts. Given an initial condition in the two-dimensional PC space, we look for the closest analogs in the historical record, as determined by their Euclidean distances in this space. We only use analogs that are at least 10 days apart, and prior to the initialization time. Then, we follow the trajectory of these historical analogs to the desired lead time, and compute the mean weighted by the inverse of the distances.

That is, starting with an initial condition in the MISO subspace $\mathbf{r}_{t_i}$, we find the closest analogs $\mathbf{r}(t_1^*)$, ..., $\mathbf{r}(t_{k_f}^*)$ in the historical record, where $t_1^*,\ldots,t_{k_f}^* < t_i$. Then, we compute the forecast $\tilde{\mathbf{r}}$ at lead time ℓ as

$$\tilde{\mathbf{r}}(t_i+\ell) = \frac{\displaystyle\sum_{j=1}^{k_f} \frac{\mathbf{r}(t_j^*+\ell)}{\|\mathbf{r}_{t_i}-\mathbf{r}(t_j^*)\|}}{\displaystyle\sum_{j=1}^{k_f} \|\mathbf{r}_{t_i}-\mathbf{r}(t_j^*)\|^{-1}}. \quad [3]$$

2. Climatological features of SEAS5

We compare the climatological features of SEAS5 to the IMD observations, to verify that it is accurate in representing the large-scale spatial and temporal features of the monsoon over India. Note that this is prior to bias correction.

Averaged over May to September, from 1993 to 2005, the SEAS5 mean rainfall in the monsoon season is 6.06 mm/day, and in IMD observations it is 5.87 mm/day. Comparing the spatial structure of seasonal monsoon rainfall between SEAS5 and observations (Fig. S2), as well as the time-series of cumulative rainfall (Fig. S3), we see that the climatologies are very similar.

3. Homogeneous rainfall regions of India

We use the homogeneous rainfall regions of India as defined in (6). The borders of the regions of India we use are shown in the map in Fig. S4.

4. Features of extracted MISO mode

Figure S5 shows the spatiotemporal structure of the extracted MISO mode for 2016. The extracted mode is almost identical to the one in (5), with the difference that the latter used May–September data as opposed to March–November here.

Figure S6 shows the average magnitude of the two-dimensional vector of PCs corresponding to the two leading empirical orthogonal functions of the MISO mode. This shows that MISO is most active from June to September, and especially in July and August.

5. Forecast skill and error metrics

Note that when averaging correlation coefficients across multiple months, in order to reduce bias, we first apply the Fisher z-transform, average, and then apply the inverse transform (7).

A. Bivariate correlation coefficient. The bivariate correlation coefficient is calculated as (8)

$$\frac{\sum_{i=1}^{N}\left[a_1(t)b_1(t,\tau)+a_2(t)b_2(t,\tau)\right]}{\left(\sum_{t=1}^{N}\left[a_1^2(t)+a_2^2(t)\right]\right)^{1/2}\left(\sum_{t=1}^{N}\left[b_1^2(t,\tau)+b_2^2(t,\tau)\right]\right)^{1/2}}, \quad [4]$$

where $a_1(t)$ and $a_2(t)$ are here the observed PC1 and PC2 at time t, $b_1(t,\tau)$ and $b_2(t,\tau)$ are the forecasted PC1 and PC2 at time t with a lead time of τ, and N is the number of samples.

B. Anomaly correlation. The uncentered anomaly correlation is computed as

$$\mathrm{AC} = \frac{\sum_{m=1}^{M}(y_m - c_m)(o_m - c_m)}{\left(\sum_{m=1}^{M}(y_m - c_m)^2 \sum_{m=1}^{M}(o_m - c_m)^2\right)^{1/2}}, \quad [5]$$

where y_m is the forecast at gridpoint m, o_m is the observation at gridpoint m, c_m is the climatological mean at gridpoint m, and M is the number of gridpoints (9). We compute c_m as the climatological mean rainfall of the same day of the year, over 30 years (1987–2016).

C. RMSE skill score. The root mean-square error (RMSE) skill score

$$1 - \frac{\mathrm{RMSE}}{\mathrm{RMSE}_{\mathrm{ref}}}, \quad [6]$$

where RMSE is the RMSE of the forecast and $\mathrm{RMSE}_{\mathrm{ref}}$ is the RMSE of a reference forecast (usually persistence or climatology) is a common skill measure for evaluating forecasts (9, 10). Here we use climatology as the reference forecast.

The RMSE skill score quantifies the skill of forecasts relative to climatology. It is higher for a better forecast, and when it reaches 0, there is no skill relative to climatology.

6. Additional evaluation of forecast skill

A. Anomaly correlation. Figure S7 compares the anomaly correlation of the uncorrected and EnOC forecasts, averaged over forecasts initialized in July, August, and September. The anomaly correlation is particularly improved in the 18–22 lead day interval, reaching an improvement of 0.034 (10%).

B. RMSE skill score. Figure S8 shows the RMSE skill score for July, August, and September. The precipitation forecasts display skill for about 22 to 23 days over both the monsoon region and India. The corrected forecasts show a higher RMSE skill score between 10 to 30 days, with an increase of up to 0.014 (Fig. S8).

C. MISO index. We examine whether there is an improvement in the forecasts' prediction of MISO. We quantify this using the bivariate real-time MISO index defined in (11).

The MISO index of the corrected forecasts is significantly improved compared to the uncorrected for July and August (Fig. S9), with an increase of up to 0.1 in the correlation coefficient at leads of 25–30 days. In September, there is not a significant change: this may be due to discrepancy in the MISO mode extracted using M-SSA and the MISO index from (11).

D. Month-by-month temporal correlation. Figure S10 shows the temporal correlations for forecasts initialized in July, August, and September.

E. Temporal correlation for weekly averaged data. Figure S11 shows the temporal correlation of forecasts initialized in July and August, but with 7-day averaging instead of 15-day averaging. Note that a 15-day window is still imposed for the intersection of the EnOC ensembles, as described in section 1A.

F. Low-level relative vorticity. Figure S12 shows the anomaly correlation between the predicted (from SEAS5) and observed (from ERA5) relative vorticity at 850 hPa. The EnOC forecasts correspond to picking the same ensemble members as those chosen for precipitation. Figure S13 shows the temporal correlation difference between the corrected and uncorrected forecasts, by region of India.

7. Statistical significance testing for skill improvements

To test whether improvements in forecast skill are statistically significant, we use bootstrap confidence intervals formed using the percentile method (12). We sample with replacement 10,000 times from years used for verification. We then compute the temporal correlation coefficient for the uncorrected (r_{uncorr}) and EnOC-corrected (r_{corr}) forecasts with respect to observations for each bootstrap sample. Then, for a given confidence level, we estimate the confidence intervals for the bootstrap distribution of $r_{\mathrm{corr}} - r_{\mathrm{uncorr}}$. If the lower limit of the interval is greater than 0, then we say that the improvement in the correlation is statistically significant.

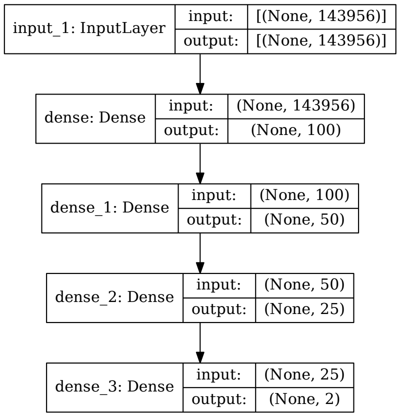

(a) Neural network for getting the initial MISO PCs

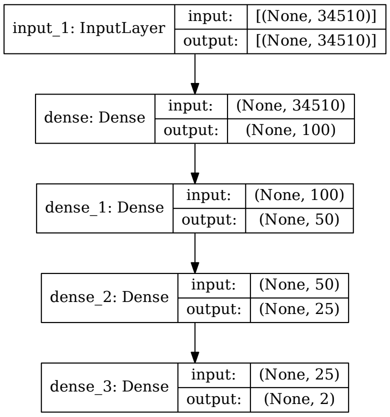

(b) Neural network for getting the ensemble member PCs

Fig. S1. Architecture of the neural networks

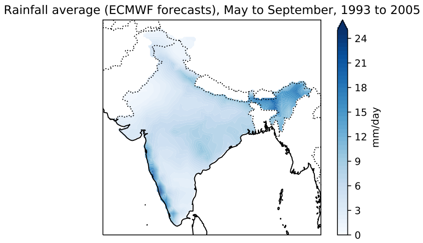

(a) SEAS5 forecasts

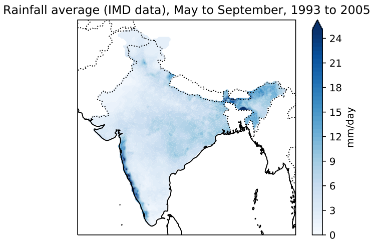

(b) IMD observations

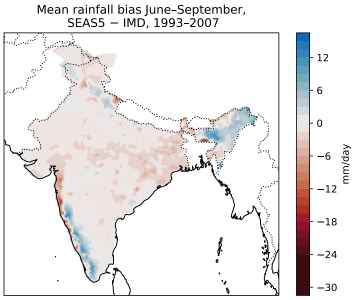

(c) Bias in mean rainfall over June to September, averaged over 1993 to 2007, comparing IMD observations and the SEAS5 hindcast ensemble mean.

Fig. S2. Climatology of average rainfall over India over the monsoon season, averaged over 1993 to 2005, comparing IMD observations and the SEAS5 hindcast ensemble mean.

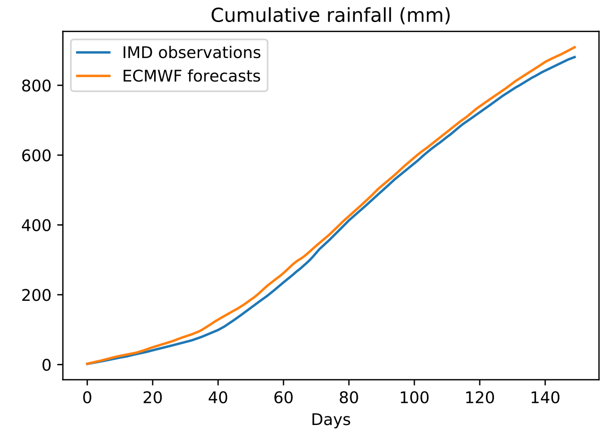

Fig. S3. Climatology of all-India cumulative rainfall over the monsoon season, averaged over 1993 to 2005, comparing IMD observations and the SEAS5 hindcast ensemble mean.

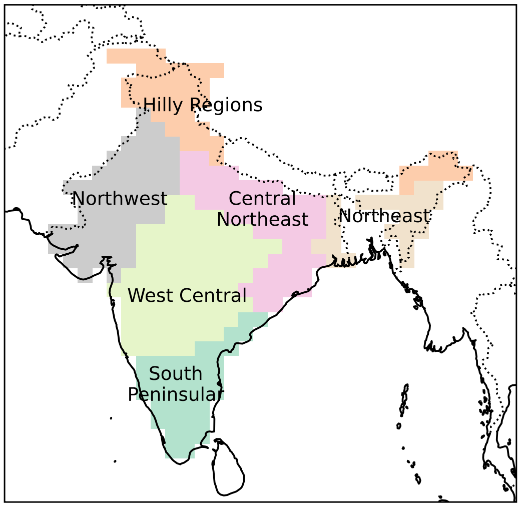

Fig. S4. Regions of India as used in the results. Note that the disputed parts of Kashmir in the north are included in the Hilly Regions region.

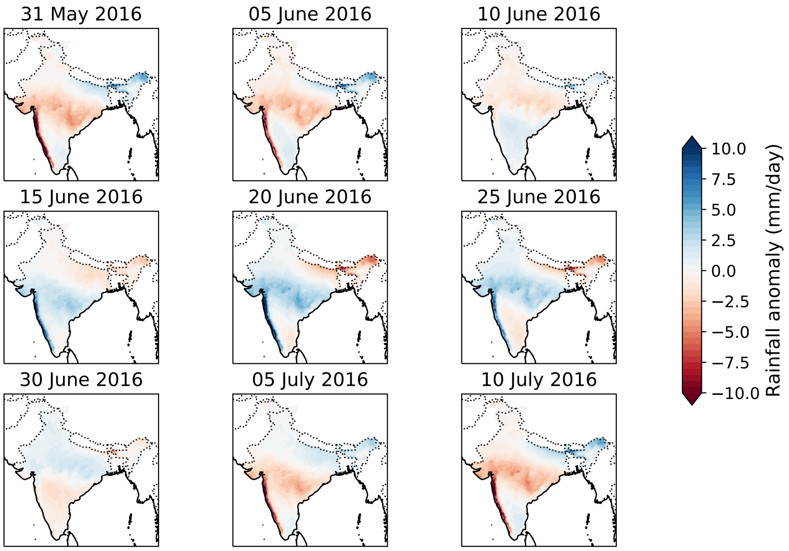

Fig. S5. The leading MISO mode from 31 May to 10 July, 2016, showing a full ~ 45-day period. Extracted from the IMD rainfall data using M-SSA.

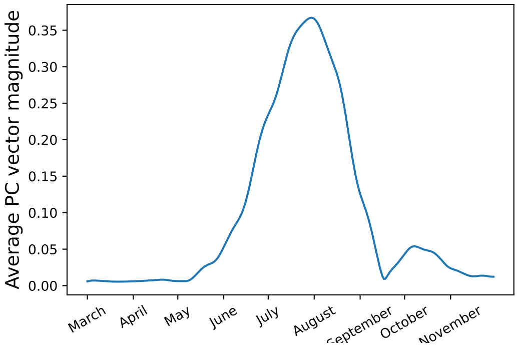

Fig. S6. Average MISO PC vector magnitude, as extracted from observations.

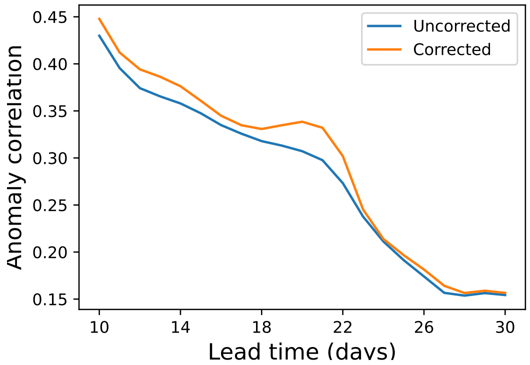

Fig. S7. Anomaly correlation of forecasted rainfall over the monsoon region.

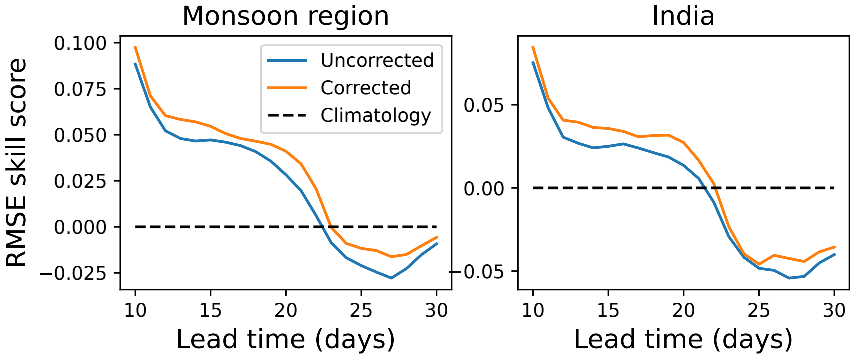

Fig. S8. RMSE skill scores of forecasted rainfall over the monsoon region and India. Higher values correspond to higher skill.

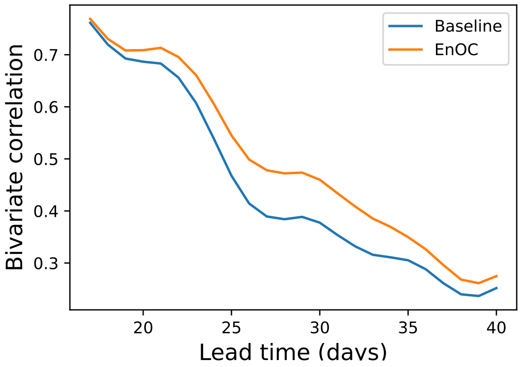

Fig. S9. MISO index correlation for forecasts initialized in July and August.

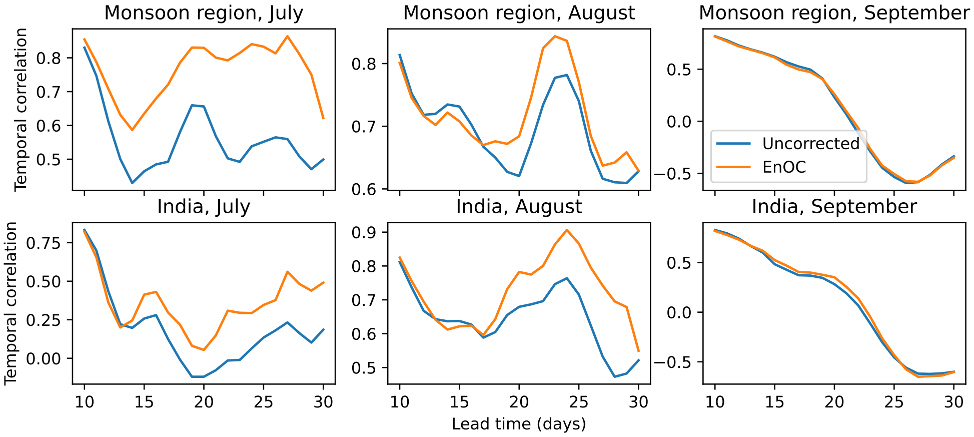

Fig. S10. Temporal correlation by month, for the monsoon region and India.

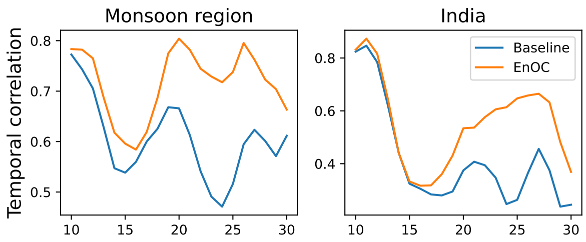

Fig. S11. Temporal correlation for forecasts initialized in July and August, with weekly averaging.

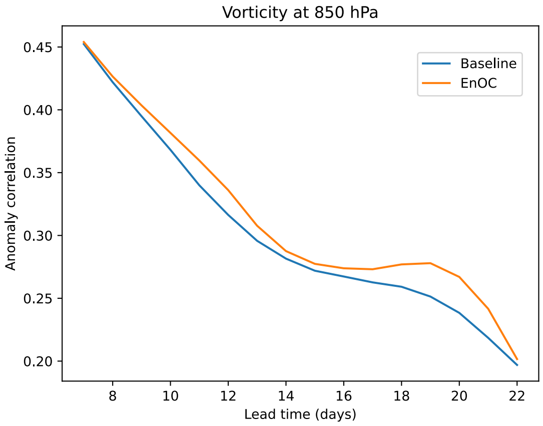

Fig. S12. Anomaly correlation of predicted relative vorticity at 850 hPa, averaged over forecasts initialized in July, August, and September.

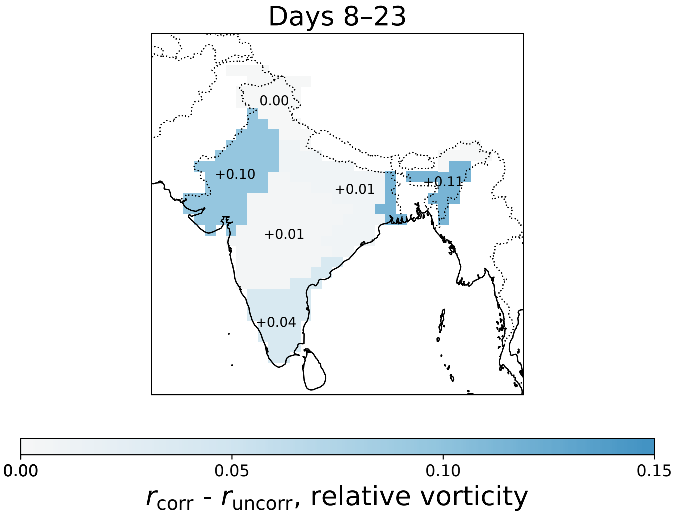

Fig. S13. Temporal correlation of predicted relative vorticity at 850 hPa, averaged over forecasts initialized in July, August, and September.

References

1. E Bach, et al., Ensemble Oscillation Correction (EnOC): Leveraging oscillatory modes to improve forecasts of chaotic systems. *J. Clim.* **34**, 5673–5686 (2021).
2. M Ghil, et al., Advanced spectral methods for climatic time series. *Rev. Geophys.* **40**, 3–1–3–41 (2002).
3. R Vautard, P Yiou, M Ghil, Singular-spectrum analysis: A toolkit for short, noisy chaotic signals. *Phys. D: Nonlinear Phenom.* **58**, 95–126 (1992).
4. M Abadi, et al., TensorFlow: Large-Scale Machine Learning on Heterogeneous Distributed Systems (2015).
5. V Krishnamurthy, AS Sharma, Predictability at intraseasonal time scale. *Geophys. Res. Lett.* **44**, 8530–8537 (2017).
6. DR Kothawale, M Rajeevan, Monthly, Seasonal and Annual Rainfall Time Series for All-India, Homogeneous Regions and Meteorological Subdivisions: 1871-2016, (Indian Institute of Tropical Meteorology, Pune, India), Research Report RR-138 (2017).
7. DM Corey, WP Dunlap, MJ Burke, Averaging Correlations: Expected Values and Bias in Combined Pearson rs and Fisher's z Transformations. *The J. Gen. Psychol.* **125**, 245–261 (1998).
8. AK Sahai, et al., Seamless Prediction of Monsoon Onset and Active/Break Phases in *Sub-Seasonal to Seasonal Prediction*, eds. AW Robertson, F Vitart. (Elsevier), pp. 421–438 (2019).
9. DS Wilks, *Statistical Methods in the Atmospheric Sciences.* (Elsevier), 4 edition, (2019).
10. AH Murphy, Skill Scores Based on the Mean Square Error and Their Relationships to the Correlation Coefficient. *Mon. Weather. Rev.* **116**, 2417–2424 (1988).
11. E Suhas, JM Neena, BN Goswami, An Indian monsoon intraseasonal oscillations (MISO) index for real time monitoring and forecast verification. *Clim. Dyn.* **40**, 2605–2616 (2013).
12. B Efron, T Hastie, *Computer Age Statistical Inference: Algorithms, Evidence, and Data Science.* (Cambridge University Press, Cambridge, U.K.), (2016).
